# Characterization and localization of side population cells in the lens

**Published:** 2010-05-29

**Authors:** Mikako Oka, Chizuko Toyoda, Yuka Kaneko, Yosuke Nakazawa, Eriko Aizu-Yokota, Makoto Takehana

**Affiliations:** Department of Molecular Function and Physiology, Faculty of Pharmacy, Keio University, Tokyo, Japan

## Abstract

**Purpose:**

Side population (SP) cells were isolated and the possibility whether lens epithelial cells contain stem cells was investigated.

**Methods:**

Mouse lens epithelial cells were stained by Hoechst 33342 and then sorted by fluorescence-activated cell sorting (FACS). The expression of stem cell markers in sorted SP cells and the main population of epithelial cells were analyzed by quantitative real-time PCR. Localization of SP cells in the mouse lens was studied by fluorescence microscopy.

**Results:**

The sorted cells contained SP cells, which were no longer separable by FACS following treatment with verapamil. The number of SP cells decreased with aging, but the adult mouse lens still contained SP cells. Phase contrast microscopy revealed that SP cells were smaller in size than non-SP cells. SP cells were localized near the equator region in lens epithelial cell layers. SP cells expressed higher levels of the stem cell markers ATP-binding cassette transporter G2 (*ABCG2*), p75 neurotrophin receptor (*p75NTR*), nestin (*nes*), B-cell lymphoma 2 (*Bcl2*), and cell surface antigen *Sca-1* mRNA than the main population cells. These results suggest that SP cells contain a high proportion of stem cells.

**Conclusions:**

The SP cells in the lens contain stem cells, and these stem cells are localized around the germinative zone.

## Introduction

Stem cells are defined as relatively undifferentiated cells and share the properties of self-renewing capacity, high proliferative potential, and multilineage differentiation capability. Embryonic stem cells are derived from blastocysts and have the ability to differentiate into all types of tissue. Somatic stem cells are undifferentiated cells found among the differentiated cells of a specific tissue or organ. They have the capacity for self-renewing and are able to differentiate into the major specialized cell types of the tissue or organ from which they were isolated. Until recently, it was believed that stem cells are not present in terminally differentiated tissues, such as the brain. However, many types of somatic stem cells have been identified, including corneal limbal stem cells in the cornea [1], hematopoietic stem cells in bone marrow, and hepatic stem cells in the liver [2,3].

Lens epithelial cells are a monolayer at the anterior surface of the lens and continue to divide and differentiate into lens fiber cells over a mammalian life. These cells divide very slowly at the germinative zone and provide fiber cells. It is consider that the stem cells of lens epithelial cells exist at or near the germinative zone [4]. Zbou et al. [5] showed by tritiated thymidine and bromodeoxyuridine (BrdU) labeling that slow-cycling cells exist at the central and germinative zones of the lens and suggested that these cells are stem cells. This process of continuous cell division suggests the existence of a population of stem cells in the lens epithelium.

One of the advantages of somatic stem cells is that there is a decreased chance of rejection following transplantation when stem cells are obtained from the recipient. However, somatic stem cells proliferate more slowly than embryonic stem cells, and surgical procedures are sometimes needed to obtain stem cells from a tissue or organ.

Side population (SP) cells are a population of stem cell-enriched cells that reportedly exist in a variety of animal tissues [6-9]. SP cells are characterized by the ability to extrude the DNA-binding dye Hoechst 33342 through the activity of ATP-binding cassette transporters [10], a feature that is common to many adult stem cells. SP cells can be separated by fluorescence-activated cell sorting (FACS) using dual-wavelength fluorescence and gating parameters for cells that display low levels of red and blue fluorescence. This method was used to identify SP cells that are highly enriched in stem cell activity [10]. In the presence of verapamil, an L-type calcium channel blocker, the stem cell activity of the isolated SP cells was abrogated.

Somatic stem cells exist among differentiated cells in an organ and can be distinguished by the expression of stem cell-specific genes called stem cell markers. Stem cell markers vary depending on the organ or tissue, although there are many markers that are present in most types of somatic stem cells. Stem cell antigen 1 (Sca-1) is a cell-surface marker for murine hematopoietic stem cells [11,12] and is also expressed in some differentiated cell types, including cells of the heart, kidney, and brain [13]. Sca-1 has been identified in bone marrow, muscle, artery, and mammary gland-derived SP cells [11,14-17]. Neurotrophin receptor p75 (p75NTR), a nerve growth factor receptor, is a stem cell marker of esophageal keratinocytes, retinal cells, and adipose tissue [18-20]. β-1 integrin is expressed at two or threefold higher levels in epidermal stem cells compared to epidermal transient amplifying cells [21], and hair follicle stem cells also express high levels of β-1 integrin [22,23]. The proto-oncogene B-cell lymphoma 2 (*Bcl2*) is an apoptosis regulatory gene and a putative epithelial stem cell marker [24,25]. Nestin (Nes) is a component of intermediate filaments and is expressed in neural progenitor cells [26]. Despite advances in the identification of somatic stem cell markers in many tissues, to date there have been no reports of stem cell markers in the lens.

In the eye, SP cells in the cornea localize to the limbal epithelium and have been shown to possess stem cell-like properties, including markedly higher expression levels of Bmi-1, Nes, Notch1, and p63 compared to non-SP cells [7,9]. Although the cells in the retina are terminally differentiated, the existence of SP cells in the retina that have stem cell properties has recently been reported [27].

To date, the existence of SP cells as stem cells in the lens has not been reported. In the current study, we investigated the presence and localization of putative lens stem cells.

## Methods

### Animals

ICR mice were purchased from Sankyo Labo Service Corporation (Tokyo, Japan). Seven-day-old mice were used, except where noted otherwise. The Keio University Animal Research Committee approved all the animal procedures used in the current study. Porcine eyes were obtained from a local slaughterhouse and were maintained on ice until use.

### Lens epithelial cell isolation

Mice were sacrificed by excess anesthesia with ether, and the eyes were enucleated to obtain the lenses. Lenses of mice were nicked under sterile conditions before immersion in a solution of trypsin. Adherent epithelial cells from the capsule were peeled away from the lens cortex. The lenses were then immersed in a solution of 0.25% trypsin-EDTA (Invitrogen Corporation, Carlsbad, CA) for 20 min to dissociate the epithelial cells from the capsules. Capsule and fiber cell debris was removed from the trypsin solution using a cell strainer (BD Biosciences, San Jose, CA), and then the epithelial cells remaining in the trypsin solution were collected by centrifugation (430× g at 4 °C for 5 min). The epithelial cells were immediately resuspended and washed three times in Dulbecco’s modified Eagle’s medium (DMEM; Invitrogen) supplemented with 10% fetal bovine serum (FBS; Cansera International Inc., Rexdale, ON, Canada) and 1% antibiotic–antimycotic (100×; Invitrogen).

### Fluorescence-activated cell sorting analysis and sorting of SP cells

Isolated lens epithelial cells were immersed in Hoechst solution (3 μg/ml Hoechst 33342; Dojindo Laboratories, Kumamoto, Japan) in DMEM, either alone or in combination with verapamil (50 µM; Sigma-Aldrich, St. Louis, MO) for 90 min at 37 °C in an atmosphere of 5% CO_2_. Immediately before FACS analysis, we added 2 µg/ml of propidium iodide (Sigma-Aldrich) to the isolated cells to distinguish live from dead cells. The analysis and separation of 10,000 lens cells was performed by FACS (BD Biosciences).

For cell-cycle analysis, isolated cells were immersed in a solution of 8 mM trisodium citrate, 2% Nonidet P-40, and 10 mg/ml propidium iodide for 30 min, on ice. Cell-cycle status was analyzed by FACS (BD Biosciences).

### Localization of side population cells in the lens epithelium

Mouse lenses were incubated in Hoechst solution for 90 min at 37 °C with or without verapamil. After staining, lenses were washed with PBS and then fixed with acetic acid:ethanol (1:3) for 10 min. Fixed lens were washed with PBS. The capsule of the lens was peeled onto a glass slide, and then the capsule with epithelial cells was mounted in a solution of 90% glycerol, 1mM p-phenylenediamine (Wako, Osaka, Japan), and 10 mM disodium hydrogen phosphate, pH 9.0.

Slides were observed by fluorescence microscopy (Leica, Weltzlar, Germany). The Hoechst blue fluorescence signals were detected by an A4 filter cassette. For the Hoechst red fluorescence signals, we constructed a special filter cassette consisting of an excitation filter (XF1005 365WB50; 365±25), a dichroic filter (XF2001 400DCLP), and an emission filter (XF3090 585ALP; Omega Optical, Inc., Brattleboro, VT) and photographed epithelial cells using a digital camera (Penguin 600CL; Pixera, San Jose CA). The fluorescence intensity of individual cells was determined using Image Master 2D platinum 7.0 (GE Healthcare UK Ltd., Little Chalfont, UK).

### Semiquantitative real-time reverse transcriptase-PCR

Total mRNA was extracted using TRIzol (Invitrogen), according to the manufacturer’s instructions, shortly, lens or cells were homogenized in TRIZOL® Reagent with glass-Teflon homogenizer. Leave the sample for 5 min at room temperature, chloroform was added to the sample. After centrifugation, the upper aqueous phase was collected. RNA was precipitated by mixing with isopropyl alcohol. The RNA pellet was washed with 75% ethanol. Primer pairs were designed using Primer Express V 2.0 software (PE Applied Biosystems, Foster City, CA). The primers for quantitative PCR were synthesized by Hokkaido System Science (Sapporo, Japan), and details of the primers and the GenBank Accession numbers are given in Table 1.

**Table 1:** Primer List for RT–PCR.

**Gene**	**Direction**	**Sequence**	**GenBank accession number**
*Actb*	Forward	5`-CACCCTGTGCTGCTCACC-3`	NM_007393
	Reverse	5`-GCACGATTTCCCTCTCAG-3`	
*ABCG2*	Forward	5`-GGAACATCGGCCTTCAAAGA-3`	NM_011920
	Reverse	5`-GCCCAATGGTTCTGAGATTCA-3`	
*β-Integrin*	Forward	5`-CTAAGTCAGCAGTGGGCACACT-3`	NM_010578
	Reverse	5`-CTCCGTCTGGCAATTTGCTATT-3`	
*p75NTR*	Forward	5`-GCCGATACGGTGACCACTGT-3`	NM_033217
	Reverse	5`-AGCCACAAGGCCCACAAC-3`	
*Bcl-2*	Forward	5`-CGTTCCTTCCTCGTCTTCCA-3`	NM_009741
	Reverse	5`-TGTGGTGAAGGGCTGTCACA-3`	
*Sca-1*	Forward	5`-TTCTCTGAGGATGGACACTTCTCA-3`	NM_010738
	Reverse	5`-AATGGGACTCCATAGCACTGGTA-3`	
*nestin*	Forward	5`-TCTTCCCCCTTGCCTAATACC-3`	NM_016701
	Reverse	5`-TTTAGGATAGGGAGCCTCAGACA-3`	

Quantitative real-time PCR was performed in triplicate in 96-well plates. Semiquantitative reverse transcriptase (RT)-PCR was performed using a One-Step SYBR® Prime Script RT-PCR Kit II (Perfect Real Time; TAKARA Bio Inc., Shiga, Japan) and a Real-Time PCR System 7300 (Applied Biosystems). Reaction conditions were based on the manufacturer’s instructions. Briefly, each 25-µl reaction consisted of 12.5 µl of one-step SYBR RT-PCR buffer 4, 2 µl of PrimeScript one-step enzyme mix 2, 2 µl of template, 1 µl of 10 pM forward and 1 µl of 10 pM reverse primers, and 0.5 µl of ROX Reference Dye (50×). The PCR amplification protocol was 42 °C for 5 min and 95 °C for 10 s, followed by 40 cycles of 95 °C for 5 s, 60 °C for 31 s, and 72 °C for 30 s. β-Actin (*Actb*) was used as an internal standard of mRNA expression.

## Results

### Fluorescence-activated cell sorting analysis of lens side population cells

**Figure 1:**
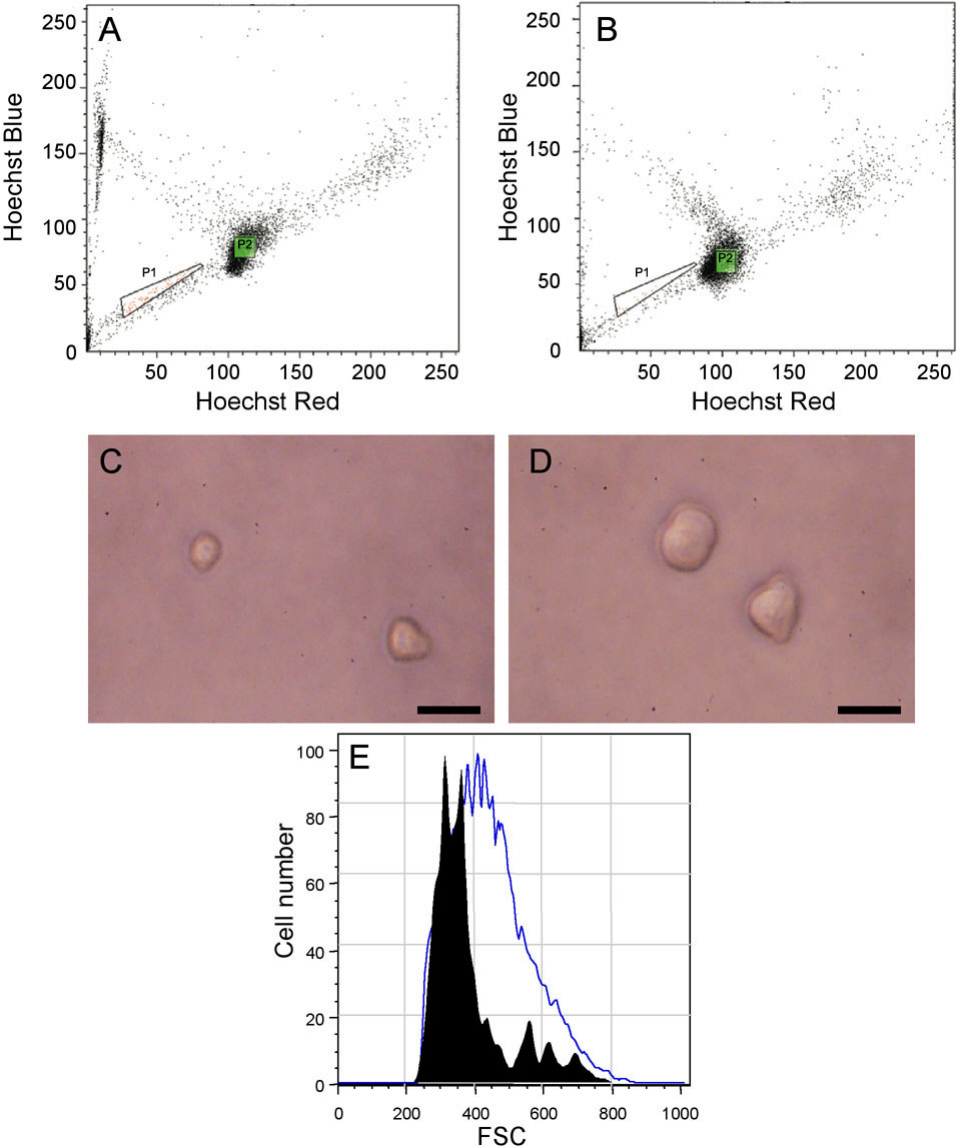
Isolation and characterization of side population (SP) cells from the mouse lens epithelium. Fluorescence-activated cell sorting (FACS) analysis of mouse lens epithelial cells stained with Hoechst 33342 alone (**A**) or in the presence of verapamil (**B**). The P1 region shows the gated region identified SP cells. The P2 region is designated as non-SP cells. SP cells (**C**) and non-SP cells (**D**) were observed by phase contrast microscopy. The scale bar represents 10 µm. (**E**) Forward scatter characteristics (FSC) of SP cells (solid area) and non-SP cells (open area) were analyzed by FACS.

We investigated the presence of putative SP cells in the lens by using FACS analysis based on the exclusion of the DNA-binding dye Hoechst 33342 in the presence and absence of verapamil. We identified SP cells that exhibited weak blue and red fluorescence (Figure 1A, P1 region). In the presence of verapamil, putative SP cells (Figure 1A, P1 region) were undetectable and could not be collected by FACS (Figure 1B, P1 region). These results confirmed that SP cells are present in the lens and can be isolated by FACS. Cells in region P2 of the FACS were designated as non-SP cells. SP and non-SP cells were collected by FACS and observed by phase contrast microscopy. SP cells were smaller in size than non-SP cells (Figure 1C, D).

Forward scatter characteristics (FSC) analysis was performed for SP and non-SP cells. A smaller FSC value means a smaller cell size. The FSC analysis of SP cells was smaller than that of non-SP cells (Figure 1E). This result confirmed that SP cells are smaller than non-SP cells.

### Cell-cycle analysis

**Figure 2:**
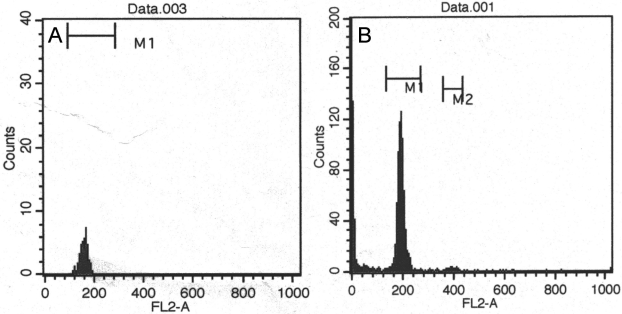
Cell-cycle analysis of side population (SP) and non-SP cells. SP (**A**) and non-SP (**B**) cells were treated with Nonidet P-40 and propidium iodide (PI), and then cell-cycle status was analyzed by FACS. The M1 region of the histogram shows cells in G_0_/G_1_ stage, and the M2 region shows cells in G_2_/M stage. The SP cell population did not contain cells in the G_2_/M stage of the cell cycle.

Somatic stem cells are predominantly in the G_1_/G_0_ stage of the cell cycle. We analyzed the cell cycle status of SP and non-SP cells by FACS following staining with propidium iodide. As shown in Figure 2, the M1 region of the histogram represents cells in G_0_/G_1_, while the M2 region represents cells in G_2_/M. All SP cells appeared to be in the G_0_/G_1_ stage of the cell cycle, and SP cells in the G_2_/M phase were undetectable (Figure 2A). Overall, the proportion of lens epithelial cells in G_2_/M was approximately 0.4% (Figure 2B). These results indicated that the cell-cycle status of SP cells in the mouse lens epithelium is similar to that of other somatic stem cells.

### Age-dependent changes in lens side population cells

**Figure 3:**
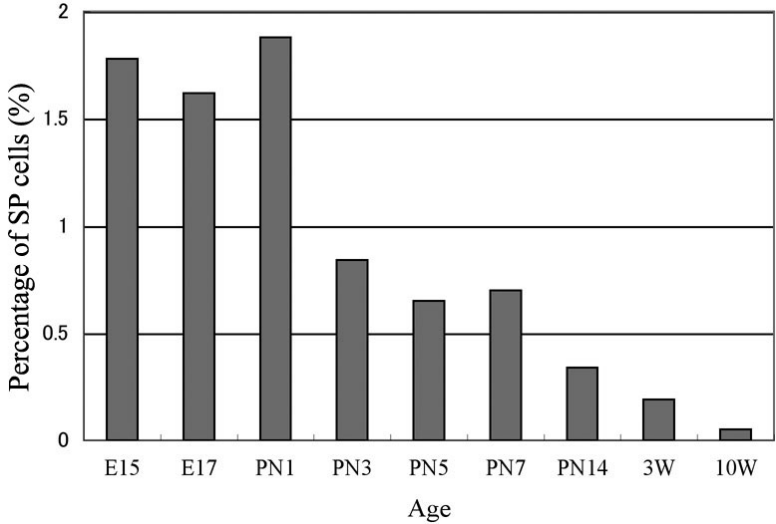
Age-dependent changes in the percentage of side population (SP) cells in the lens epithelium. The percentage of SP cells was measured at embryonic day 15 (E15), E17, postnatal day 1 (PN1), PN3, PN5, PN7, PN14, 3 weeks of age (3W), 10W. The percentage of SP cells decreased at PN3 in mice and was approximately 0.05% in mice at 10W.

We investigated the percentage of SP cells in the lens at different ages. Epithelial cells in the lenses of embryonic mice contained a high percentage of SP cells, approximately 1.9% (Figure 3). After birth, the percentage of SP cells in the lens epithelium decreased, and by 10 weeks of age, the percentage of SP cells in the mouse lens was approximately 0.05%.

### Localization of side population cells in the lens epithelium

**Figure 4:**
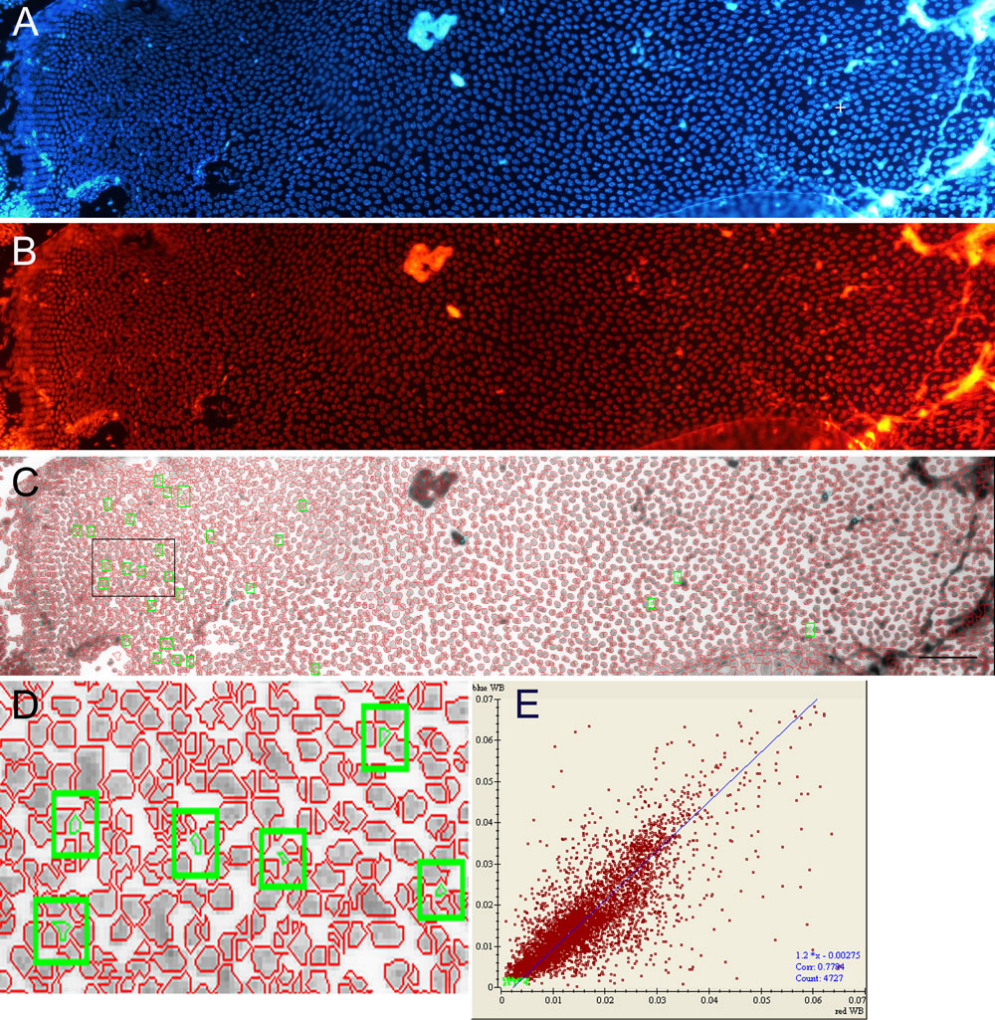
Localization of side population (SP) cells in the lens epithelium. Mouse lenses were incubated in DMEM containing Hoechst 33342 for 90 min and then fixed. **A** shows Hoechst blue fluorescent image and **B** shows Hoechst red fluorescent image of the same mouse lens epithelium. The “+” in (**A**) indicates the center of the lens epithelium. **C** shows the result of fluorescent intensity analysis using Image Master software. The cells with the lowest levels of fluorescence intensity, shown in the green spot in **E,** can be seen as green cells highlighted with a green square in **C**. **D** shows high magnification images of the cells within the areas marked by black square in **C**. SP cells localized around the germinative zone of the lens epithelium. The scale bar represents 100 µm. **E** shows a plot of the cells at the indicated levels of intensity of Hoechst blue (vertical axis) and red fluorescence (horizontal axis).

To determine the localization of SP cells in the lens epithelium, mouse lenses were incubated in DMEM containing Hoechst 33342 for 90 min and then fixed. Capsules from the fixed lenses were carefully peeled onto a glass slide. Figure 4A shows a representative blue fluorescent image of lens epithelial cells on a peeled capsule stained with Hoechst 33342. The same area of the epithelium from the same mouse lens was also observed using a filter cassette for red fluorescence (Figure 4B). Using Image Master software, we analyzed the fluorescence intensity of individual cells. The area that Image Master recognized as a cell is shown with a red circle (Figure 4C). Figure 4D shows a higher magnification image of the cells surrounded by a square in Figure 4C. Figure 4E shows a plot of the cells at the indicated levels of intensity of Hoechst blue (vertical axis) and red fluorescence (transverse axis). Cells with the lowest fluorescence intensity are indicated by a green spot and are visible as green cells highlighted with a green square in Figure 4C. Based on this analysis of Hoechst blue and red fluorescence, 42 cells with the lowest levels of fluorescence intensity were selected for total cell number 6292; these are visible as green cells in Figure 4C. These results demonstrated that SP cells, characterized by low Hoechst fluorescence intensity (both blue and red) mainly localize around the germinative zone of the lens epithelium. The cells selected for this analysis were smaller than the other cells and were close to the next cell. Similar results were obtained from three different samples.

**Figure 5:**
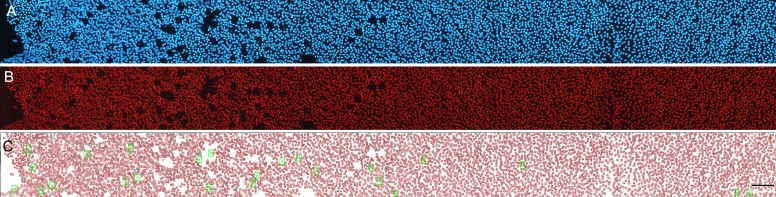
Localization of side population (SP) cells in the porcine lens. The fluorescent images of Hoechst blue (**A**) and red (**B**) fluorescence in the porcine lens epithelium were analyzed using Image Master software. **C**: Cells with the lowest levels of blue and red fluorescence are visible as green cells. The scale bar represents 200 µm.

We performed a similar analysis of SP cell localization in porcine lenses (Figure 5). The results indicated that SP cells in the porcine lens were also few at the center of the epithelium and localized around the germinative zone. Thirty-one SP cells showed as green cells highlighted by green squares, and 5690 non-SP cells recognized by Image Master were indicated as red-circled cells.

### Expression of stem cell markers in lens side population cells

**Figure 6:**
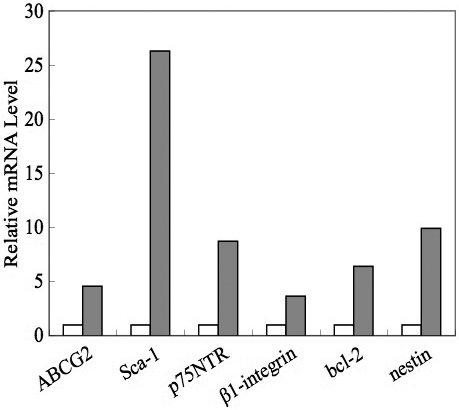
Semiquantitative reverse transcriptase (RT)-PCR analysis. The expression of the stem cell markers ATP-binding cassette transporter G2 (*ABCG2*), cell surface antigen Sca-1, p75 neurotrophin receptor (*p75NTR*), β-1 integrin, B-cell lymphoma 2 (*Bcl2*), and nestin in SP and non-SP cells was analyzed by semiquantitative RT-PCR. Open bars indicate mRNA levels in non-SP cells; gray bars indicate mRNA levels in SP cells. SP cells express more *ABCG2* than non-SP cell. All stem cell markers were expressed at higher levels in SP cells than non-SP cells. Stem cell antigen 1 was expressed at 26 fold higher levels in SP cells than in non-SP cells.

SP and non-SP cells were isolated from the mouse lens epithelium and subjected to real-time semiquantitative RT-PCR analysis (Figure 6). SP cells expressed approximately 4.5-fold higher mRNA levels of the stem cell marker *ABCG2* compared to non-SP cells, which strongly suggested that mouse lens SP cells isolated by FACS are stem cells. We also analyzed the expression of the common stem cell markers *Sca-1*, *p74NTR*, β-1 integrin (*Itgb1*), *Bcl-2*, and *Nes* in SP and non-SP cells. All of these stem cell markers were expressed at much higher levels in SP cells than in non-SP cells. This was particularly true for *Sca-1* (a cell surface marker for murine hematopoietic stem cells), which was expressed at 26-fold higher levels in SP cells than in non-SP cells (Figure 6).

## Discussion

Throughout the life of the animal, lens epithelial cells continue to divide and differentiate into lens fiber cells. This process of continuous cell division suggests the existence of stem cells in the lens epithelium. The lens is covered with a capsule, which prevents the inward migration of stem cells from outside the lens. Therefore, it has been shown by labeling with tritiated thymidine and BrdU or stem cell marker [4,5] that the stem cell of lens epithelial cells is at or near the germinative zone.

In the current study, we showed that lens epithelial cells contain SP cells and that these cells share some of the same properties as stem cells. These results raise the question of whether SP cells isolated from lens epithelial cells are true stem cells. In many other organs, stem cell-enriched populations of cells termed SP cells have been identified based on their ability to extrude Hoechst 33342 dye. In general, treatment with the calcium channel blocker verapamil completely abrogates this characteristic property of SP cells. In the current study, we observed the same phenomenon in SP cells isolated from lens epithelial cells. Real-time semiquantitative RT-PCR showed that lens epithelial SP cells express higher levels of *ABCG2* than non-SP cells. SP cells were smaller than non-SP cells, which is another property of stem cells [7]. Furthermore, stem cell markers that are present in stem cells from other tissues, such as *Sca-1*, *p75NTR*, and *Nes*, were highly expressed in lens epithelial SP cells.

The proportion of lens epithelial SP cells decreased rapidly from birth to 3 days of age in mice. The same phenomenon has been reported in the retina, heart, and skin [22,27,28]. The percentage of retinal SP cells is 0.1% during embryogenesis and decreases dramatically at birth, reaching a low steady-state level in the adult retina [19]. Newborn mouse skin also contains a high percentage of SP cells, which decreases with age [22,28,29]. Our results demonstrated that the percentage of SP cells in the lens epithelium also decreases rapidly after birth but that SP cells exist at low levels in the adult mouse lens epithelium. This result was suggested by semiquantitative RT-PCR.

Cell-cycle analysis revealed that all lens epithelial SP cells were in the G_0_/G_1_ phase of the cell cycle and that SP cells in G_2_/S were undetectable. These results strongly suggest that SP cells isolated from lens epithelial cells are stem cells.

Using fluorescence microscopy and two filter sets that were specific for Hoechst 33342 blue and red fluorescence, we analyzed the location of SP cells in the mouse lens. Following Hoechst staining, lenses were photographed using a digital camera, and then the images were analyzed using Image Master software. SP cells in the lens epithelium localized around the germinative zone and were undetectable in the central region. Stem cells are believed to exist in a special microenvironment termed *niche*. The niche provides signals to the stem cells that regulate self-renewal, cell division, and differentiation [30]. Lens epithelial cell division occurs at the germinative zone. Our results suggest that lens stem cells located around the germinative zone are the source of dividing cells in this area.

The stem cell niche is a specific microenvironment in which adult somatic stem cells reside within their tissue of origin. The niche is needed to maintain the self-renewing capacity of stem cells as well as their multilineage potential. Separation from the niche compartment induces stem cell differentiation. In general, the niche is believed to be a highly organized microenvironment in which a variety of factors, including secreted cytokines, extracellular matrix interactions, and cell–cell adhesion, function cooperatively to maintain the undifferentiated stem cell phenotype [31]. β integrins are present in many organs and function in the attachment of cells to the extracellular matrix. In stem cells, β integrins are thought to maintain the microenvironment of the basement membrane. We showed that β-1 integrin is expressed in SP cells at much higher levels than in non-SP cells, which suggests that β-1 integrin maintains the stem cell phenotype of SP cells in the lens and thus the source of dividing cells in the lens.
